# Spontaneous Heterotopic Pregnancy: A Case Report of a Rare and Critical Obstetric Condition

**DOI:** 10.7759/cureus.77020

**Published:** 2025-01-06

**Authors:** Merfat M Flmban, Reem H Mohammed, Hanan M Alshehri, Suhad A Aljuhani, Rahaf F Baamer, Nouf A Alsahafi, Alaa M Aljedani

**Affiliations:** 1 Obstetrics and Gynecology, Al Thagher General Hospital, Jeddah, SAU; 2 General Surgery, Al Thagher General Hospital, Jeddah, SAU; 3 Obstetrics and Gynaecology, East Jeddah Hospital, Jeddah, SAU

**Keywords:** ectopic pregnancy, heterotopic pregnancy, laparoscopy, salpingectomy, spontaneous pregnancy

## Abstract

Heterotopic pregnancy is defined as the concurrent presence of both an intrauterine pregnancy and an extrauterine (typically ectopic) pregnancy. This report presents the case of a 36-year-old female patient who presented to the emergency department with lower abdominal pain. A comprehensive evaluation, including transabdominal and transvaginal ultrasound imaging, revealed a heterotopic pregnancy at an estimated gestational age of six weeks and two days. The ultrasound examination confirmed an intrauterine pregnancy with fetal cardiac activity and a visible fetal pole, as well as a complex vascular lesion in the left adnexal region. Consequently, the patient underwent exploratory laparotomy, including a left salpingectomy, to remove the ruptured left tubal ectopic pregnancy. The ectopic pregnancy was successfully excised, and the intrauterine embryo was preserved. Following this surgical intervention, the pregnancy progressed without complications, culminating in the delivery of a healthy infant at 39 weeks of gestation. With timely and appropriate treatment, a heterotopic pregnancy can result in a successful live birth, yielding favorable outcomes for both mother and child. This case underscores the necessity of considering heterotopic pregnancy even in the absence of risk factors and highlights the importance of multidisciplinary management to achieve optimal outcomes.

## Introduction

Heterotopic pregnancy involves the simultaneous presence of both an intrauterine and an extrauterine pregnancy, most commonly located in the fallopian tube, though it may also occur in the cervix or ovary. The incidence of spontaneous heterotopic pregnancy in the general population is approximately one in 30,000 cases [1].

The incidence is notably higher in women undergoing assisted reproductive technologies, such as in vitro fertilization (IVF), reaching as high as one in 3,900. Additional risk factors for heterotopic pregnancy parallel those for ectopic pregnancy, including a history of pelvic inflammatory disease (PID), previous abdominal or pelvic surgery, uterine anomalies, use of intrauterine devices, smoking, and in utero exposure to diethylstilbestrol (DES) [2].

Patients with heterotopic pregnancy often present with pelvic pain, vaginal bleeding, and amenorrhea, although they may be asymptomatic or exhibit nonspecific symptoms [3]. Severe cases can present with hemodynamic instability due to rupture of the ectopic pregnancy. The clinical presentation can resemble a threatened abortion or an isolated ectopic pregnancy, complicating early diagnosis.

Measurement of serum beta-human chorionic gonadotropin (β-hCG) and ultrasound examination are critical for diagnosis. Irregularly elevated β-hCG levels that do not match the expected intrauterine gestational age warrant further investigation. Transvaginal ultrasound (TVUS) improves diagnostic accuracy, although its sensitivity is only about 56% at 5-6 weeks of gestation [4].

We present a case of heterotopic pregnancy in a 36-year-old patient, emphasizing the diagnostic challenges and the successful outcome following timely surgical intervention.

## Case presentation

A 36-year-old patient, G6P4+1, presented with an estimated gestational age of four weeks and six days based on her last menstrual period (LMP). She had no significant medical or surgical history. She arrived at the emergency department complaining of lower abdominal pain for one day, described as intermittent, non-radiating, partially relieved by analgesics, and aggravated by standing and walking. She also reported dysuria and urinary frequency but denied nausea and vomiting.

**Figure 1:**
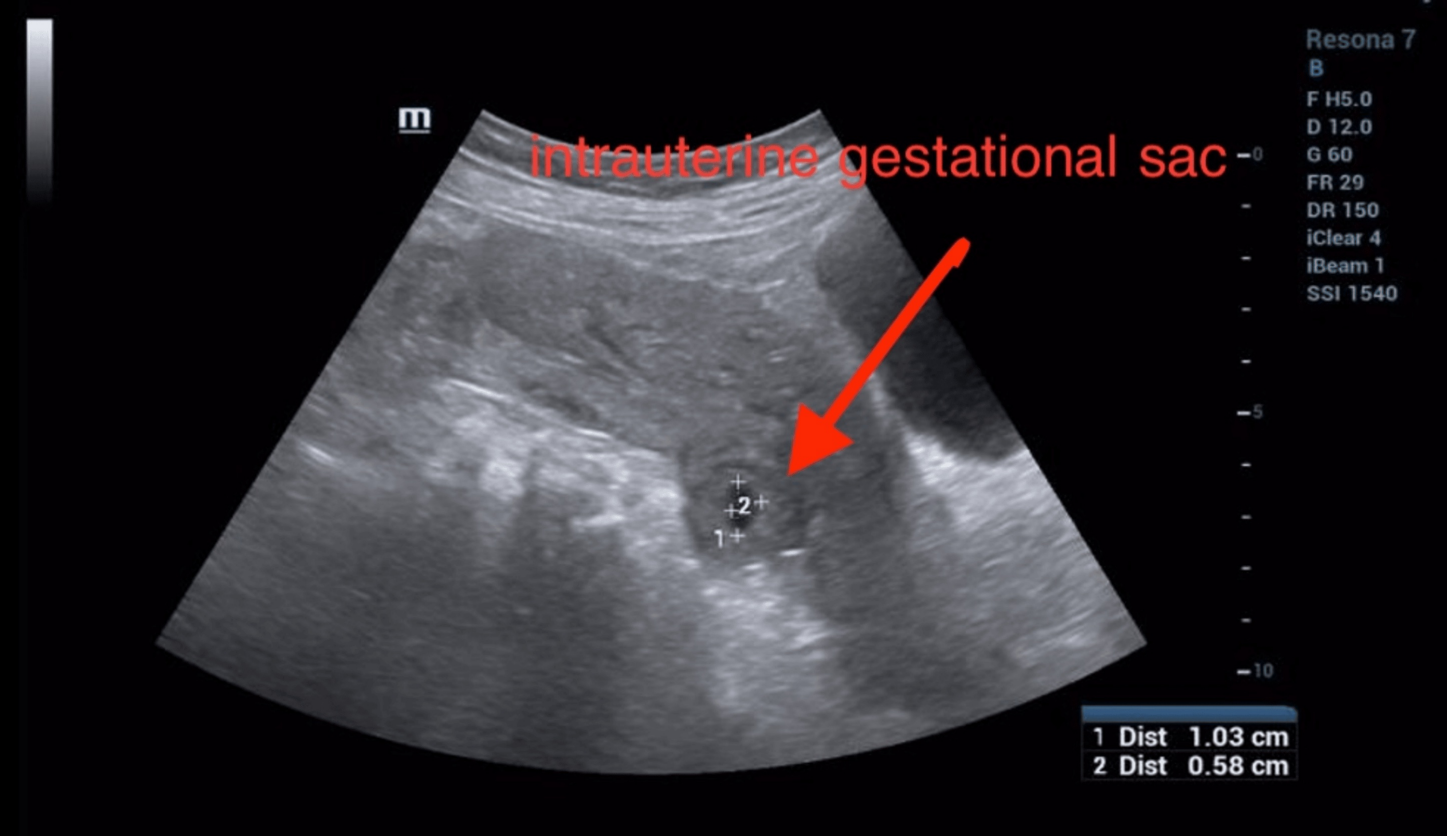
Transabdominal ultrasonography image of uterus shows a single empty gestational sac seen in the uterine cavity.

On physical examination, the patient was hemodynamically stable, revealing a soft, lax abdomen with mild tenderness on deep palpation and no rebound tenderness. Initial laboratory tests showed hemoglobin (Hb) of 8.6 g/dL, a white blood cell count (WBC) of 16 × 10^9^/L, and a β-hCG level of 17,458 mIU/mL. Ultrasonography revealed a gravid uterus with an empty gestational sac (Figure 1). Free fluid in the right iliac fossa and minimal intraperitoneal fluid raised suspicion of appendicitis or a ruptured ovarian follicle. She was diagnosed with early pregnancy and a presumed ruptured ovarian cyst, then discharged with antibiotics and analgesics for outpatient follow-up.

Two days later, she returned to the emergency department with ongoing abdominal pain, new-onset vaginal spotting, and persistent dysuria. She remained hemodynamically stable. A bedside ultrasound showed an intrauterine gestational sac but no fetal pole, and no extrauterine sac was detected. Vaginal examination revealed a closed cervix with minimal bleeding and no cervical motion tenderness. The plan was to repeat β-hCG, but the patient left against medical advice. The β-hCG result later returned at 21,000 mIU/mL.

**Figure 2:**
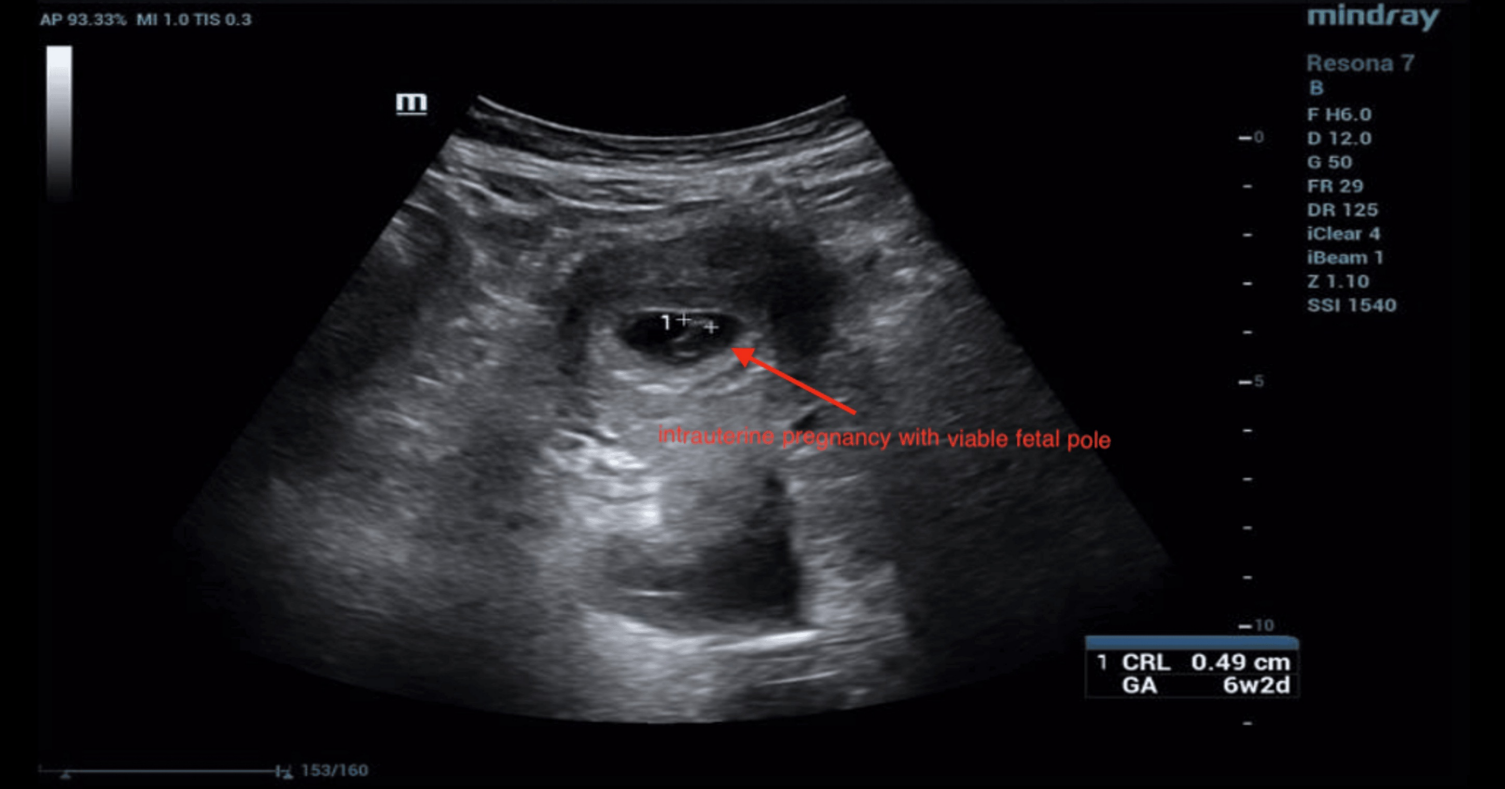
Transabdominal ultrasonography image of uterus shows a single gestational sac seen in the uterine cavity containing single viable fetal pole, corresponding to six weeks and two days, with a crown-rump length of 0.49 cm.

**Figure 3:**
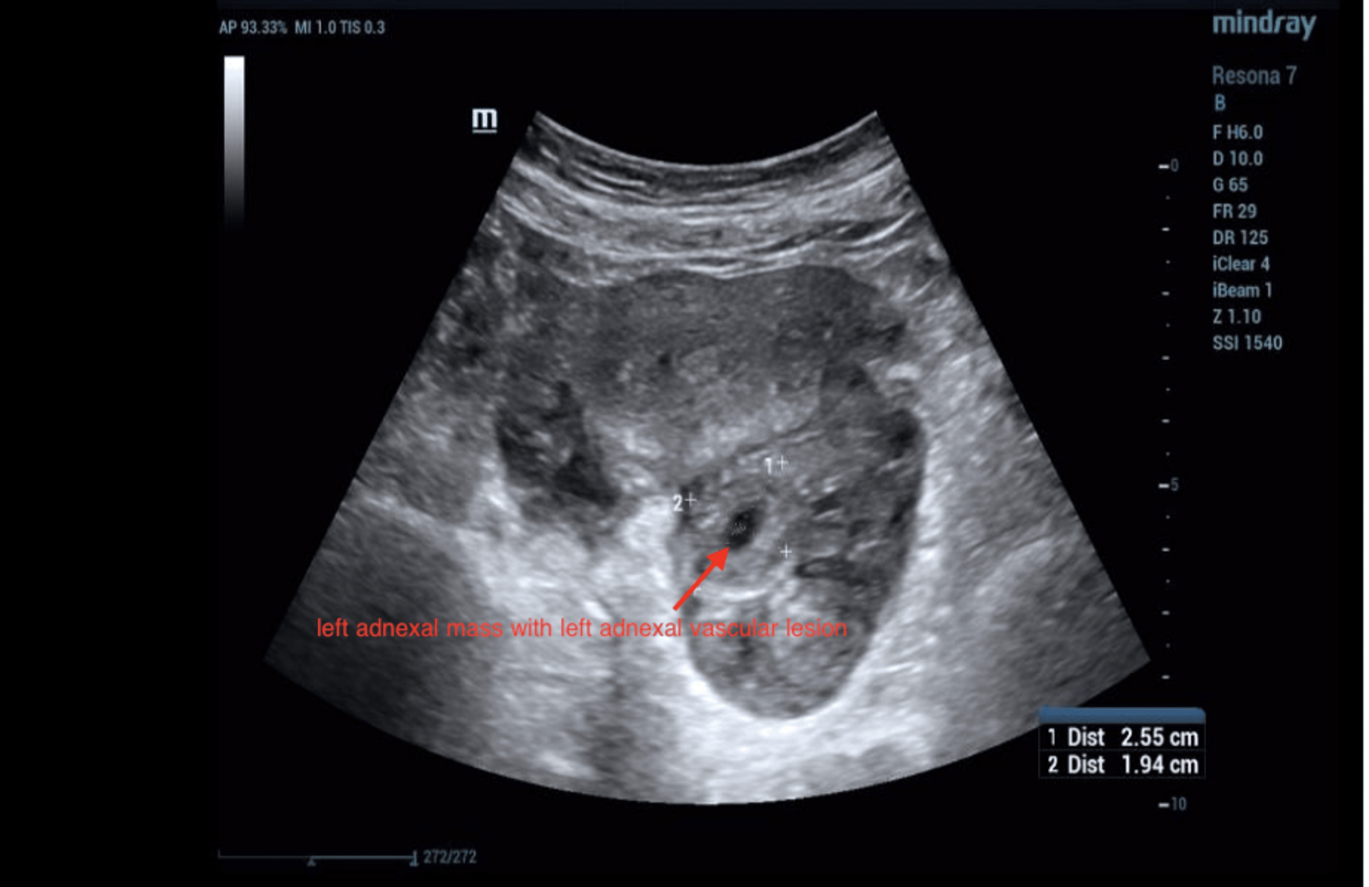
Transabdominal ultrasonography image of uterus shows a left adnexal mass with left adnexal vascular lesion (complicated left ectopic gestation).

One week later (approximately six weeks of gestation), the patient presented to the outpatient clinic with similar complaints. Laboratory tests showed a WBC of 10.9 × 10^9^/L, an Hb of 7.99 g/dL, and a β-hCG level of 64,800 mIU/mL (Table 1). Repeat ultrasonography revealed a gravid uterus containing a single viable fetus measuring six weeks and two days (Figure 2), accompanied by a complex left adnexal mass and a left adnexal vascular lesion (Figure 3). The differential diagnoses included left ectopic pregnancy, left tubo-ovarian abscess, or complicated appendicitis.

**Table 1:** Laboratory results over three visits with corresponding reference ranges and visit timelines. Hb: hemoglobin; WBC: white blood cell; β-hCG: beta-human chorionic gonadotropin.

Test	Initial visit	Second visit (Day 2)	Third visit (Day 9)	Normal reference range
Hb	8.6 g/dL	Not performed	7.99 g/dL	12-15.5 g/dL (women)
WBC count	16 × 10^9^/L	Not performed	10.9 × 10^9^/L	4-11 × 10^9^/L
β-hCG	17,458 mIU/mL	21,000 mIU/mL	64,800 mIU/mL	Varies with gestation age: less than 5 mIU/mL (non-pregnant)

The patient was admitted for a diagnostic laparoscopy with a multidisciplinary team including general surgery and gynecology. Diagnostic laparoscopy revealed a normal appendix and a ruptured left tubal ectopic pregnancy consistent with a heterotopic pregnancy. A left salpingectomy was performed, and the patient was transferred to the ward in good condition. The following day, after confirming a viable intrauterine pregnancy on ultrasound, she was discharged with instructions to take folic acid, paracetamol (1 g as needed), and nitrofurantoin. Her antenatal follow-up was uneventful. She ultimately delivered a healthy infant at 39 weeks and three days of gestation without complications.

## Discussion

Spontaneous heterotopic pregnancy, defined as the concurrent presence of an intrauterine and an extrauterine gestation, is a rare but clinically significant diagnostic challenge. Although the incidence of heterotopic pregnancy has increased with the use of assisted reproductive technologies, it can also occur spontaneously in the absence of known risk factors.

Clinically, heterotopic pregnancy can mimic a miscarriage or an isolated ectopic pregnancy. Common presentations include lower abdominal pain, adnexal masses, and signs of peritoneal irritation. Thus, it can be difficult to distinguish heterotopic pregnancy from other gynecological conditions, including isolated ectopic pregnancy and spontaneous abortion [5].

All pregnant women presenting with abdominal pain or vaginal bleeding should be thoroughly evaluated. Ultrasound imaging and serial β-hCG measurements are essential for accurately localizing the pregnancy. Identifying an intrauterine pregnancy does not exclude the possibility of a concurrent ectopic gestation [6].

The mainstay treatment of heterotopic pregnancies requires consideration of the patient's hemodynamic stability, prognosis or desired outcome, and the site of implantation of ectopic pregnancy. If the patient is hemodynamically stable, laparoscopy is preferred over laparotomy as it is less invasive. The first line of treatment is salpingectomy to remove the extrauterine pregnancy, but it is vital to ensure proper methods are used to preserve the existing intrauterine pregnancy. If the intrauterine pregnancy is desired and the patient is hemodynamically stable, the patient can undergo transvaginal injection of feticidal substances, such as potassium chloride or hyperosmolar glucose, which are often tolerated by the intrauterine pregnancy [7].

In this case, the patient initially presented with nonspecific symptoms and no identified risk factors. Serial β-hCG measurements and repeated ultrasound examinations were crucial in eventually diagnosing the heterotopic pregnancy. Surgical intervention (left salpingectomy) promptly addressed the ectopic component while preserving the intrauterine pregnancy, enabling a successful full-term delivery.

This case underscores the importance of multidisciplinary care in achieving successful outcomes. The diagnostic process was initiated by the junior clinician, who promptly escalated the case to the senior clinician, who then referred it to the obstetrician-gynecologist consultants. Recognizing the complexity of the situation, the consultants engaged the general surgery consultant to address the differential diagnosis. The surgical intervention was led by the obstetricians, with critical support from the general surgeon and the assisting team. Postoperative care and follow-up were managed collaboratively by the senior clinicians and the junior clinician, ensuring comprehensive monitoring and a positive outcome. This teamwork exemplifies the value of a multidisciplinary approach in managing complex clinical situations.

## Conclusions

Spontaneous heterotopic pregnancy, though rare, is a potentially life-threatening condition that requires a high index of suspicion. This case highlights the importance of considering heterotopic pregnancy in patients presenting with abdominal pain and vaginal bleeding, even in the absence of risk factors. Early and accurate diagnosis, supported by transvaginal ultrasound and serial β-hCG measurements, can reduce maternal morbidity and facilitate the successful preservation of the intrauterine pregnancy. Timely surgical management remains the cornerstone of treatment.
